# The Periodontal Pathogen *Fusobacterium nucleatum* Exacerbates Alzheimer’s Pathogenesis *via* Specific Pathways

**DOI:** 10.3389/fnagi.2022.912709

**Published:** 2022-06-23

**Authors:** Hongle Wu, Wei Qiu, Xiaofang Zhu, Xiangfen Li, Zhongcong Xie, Isabel Carreras, Alpaslan Dedeoglu, Thomas Van Dyke, Yiping W. Han, Nadeem Karimbux, Qisheng Tu, Lei Cheng, Jake Chen

**Affiliations:** ^1^Department of Endodontics, Stomatological Hospital, Southern Medical University, Guangzhou, China; ^2^State Key Laboratory of Oral Disease, West China Hospital of Stomatology, National Clinical Research Center for Oral Diseases, Sichuan University, Chengdu, China; ^3^Division of Oral Biology, Tufts University School of Dental Medicine, Boston, MA, United States; ^4^Department of Stomatology, Nanfang Hospital, Southern Medical University, Guangzhou, China; ^5^Department of Periodontology, Tufts University School of Dental Medicine, Boston, MA, United States; ^6^Geriatric Anesthesia Research Unit, Department of Anesthesia, Critical Care and Pain Medicine, Massachusetts General Hospital and Harvard Medical School, Charlestown, MA, United States; ^7^Department of Veterans Affairs, VA Boston Healthcare System, Boston, MA, United States; ^8^Department of Neurology and Department of Biochemistry School of Medicine, Boston University, Boston, MA, United States; ^9^Department of Veterans Affairs, VA Boston Healthcare System, Boston, MA, United States; ^10^Department of Neurology School of Medicine, Boston University, Boston, MA, United States; ^11^The Forsyth Institute, Clinical and Translational Research, Cambridge, MA, United States; ^12^Department of Oral Medicine, Infection, and Immunity, Harvard School of Dental Medicine, Boston, MA, United States; ^13^Section of Oral, Diagnostic and Rehabilitation Sciences, College of Dental Medicine, University Irvign Medical Center, New York, NY, United States; ^14^Department of Microbiology & Immunology, Vagelos College of Physicians & Surgeons, Columbia University Irvign Medical Center, New York, NY, United States; ^15^Department of Developmental, Molecular and Chemical Biology, Tufts University School of Medicine, Boston, MA, United States; ^16^Graduate School of Biomedical Sciences, Tufts University, Boston, MA, United States

**Keywords:** *F. nucleatum*, inflammation, periodontitis, Alzheimer’s Disease, mouse model

## Abstract

Alzheimer’s Disease (AD) is the most common form of dementia in older adults and has a devastating impact on the patient’s quality of life, which creates a significant socio-economic burden for the affected individuals and their families. In recent years, studies have identified a relationship between periodontitis and AD. Periodontitis is an infectious/inflammatory disease that destroys the supporting periodontal structure leading to tooth loss. Dysbiosis of the oral microbiome plays a significant role in the onset and development of periodontitis exhibiting a shift to overgrowth of pathobionts in the normal microflora with increasing local inflammation. *Fusobacterium nucleatum* is a common pathogen that significantly overgrows in periodontitis and has also been linked to various systemic diseases. Earlier studies have reported that antibodies to *F. nucleatum* can be detected in the serum of patients with AD or cognitive impairment, but a causal relationship and a plausible mechanism linking the two diseases have not been identified. In this study, we conducted both *in vivo* and *in vitro* experiments and found that *F. nucleatum* activates microglial cells causing morphological changes, accelerated proliferation and enhanced expression of TNF-α and IL-1β in microglial cells. In our *in vivo* experiments, we found that *F. nucleatum*-induced periodontitis resulted in the exacerbation of Alzheimer’s symptoms in 5XFAD mice including increased cognitive impairment, beta-amyloid accumulation and Tau protein phosphorylation in the mouse cerebrum. This study may suggest a possible link between a periodontal pathogen and AD and *F. nucleatum* could be a risk factor in the pathogenesis of AD. We are currently further identifying the pathways through which *F. nucleatum* modulates molecular elements in enhancing AD symptoms and signs. Data are available *via* ProteomeXchange with identifier PXD033147.

## Introduction

Alzheimer’s Disease (AD) is the most common cause of dementia in older adults. Patients suffer from cognitive impairment and memory loss, and in later stages, language and visuospatial dysfunction seriously impact daily activities and quality of life (Lane et al., 2018; Weuve et al., 2018; Jia et al., 2020). Complications of AD include skin infections, organ failure, blood clots and other problems that can be fatal. AD is the main cause of dependency in the elderly over 65, which has created a huge public health burden (Jia et al., 2018, 2020). In United States, among people over 65, about 6.5 million suffer from AD in 2022 (Hebert et al., 2013; Alzheimer’s Association Report, 2022). The prevalence of this disease increases significantly with age; reaching nearly 33.2% in the elderly over the age of 85 (Alzheimer’s Association Report, 2022). With the increase in population age and life expectancy, the prevalence of AD will further increase, and it is expected to affect approximately 14 million people by 2060 in the United States (Abbayya et al., 2015; Alzheimer’s Association Report, 2022).

The main pathological manifestation of AD is senile plaque formation by abnormally folded beta-amyloid (Aβ) protein outside of neurons and neurofibrillary tangles. The proposed cause of Aβ plaques is hyperphosphorylated Tau protein inside neurons (Ando et al., 2020; Coomans et al., 2021), which leads to neurodegeneration in the brain (Karran et al., 2011; Yu et al., 2020). In addition, microbiota of host may also be a key factor that might influence the accumulation of beta-amyloid. Wang et al. (2015) found that intestinal microbiota GSPE metabolites could interfere the Aβ assembly by enter into the brain. “Microbiota-gut-brain axis” is a significant theory which tells us the close relationship between gut microbiota and brain function (Liu et al., 2020a; Doifode et al., 2021). Oral mictobiota as the indispensable part of host microbiome, more and more researchers put an eye on its impact on Alzheimer’s Disease and found the possible role that oral microbe could play on the onset and progress of Alzheimer’s Disease. However, the exact pathogenesis of AD is still unclear. Age, genetics and having a family history are the greatest risk factors for Alzheimer’s Disease (2020).

Inflammation is a key factor in the pathogenesis of AD. Pro-inflammatory cytokines produced by various immune-associated cells, including activated microglia, can further auto-stimulate through paracrine and/or autocrine pathways leading to increases in β-amyloid, p -Tau and pro-inflammatory molecules and finally, neurodegeneration (Akiyama et al., 2000; Cai et al., 2014). Here, we investigate microglia as they play a significant role in the progression of AD.

Periodontitis is the most common oral inflammatory disease and is the single greatest cause of adult tooth loss (Kinane et al., 2017). Periodontitis has a major impact on quality of life, but also poses a threat to the patient’s overall health (Eke et al., 2012). A number of studies describe a link between periodontitis and systemic diseases (Sampaio-Maia et al., 2016), including diabetes (Chapple et al., 2013), cardiovascular disease (Dietrich et al., 2013; Tonetti et al., 2013), respiratory disease, adverse pregnancy outcomes (He et al., 2015), cancer and nervous system diseases (Noble et al., 2009; Perera et al., 2016). In recent years, there have been an increasing number of studies focused on the association between periodontitis and AD suggesting that periodontitis can promote the initiation and progression of AD (Kantarci et al., 2020; Liccardo et al., 2020). Through a population-based retrospective matched-cohort study, Chen et al. found that having periodontitis for 10 or more years increased the risk of AD (Odds Ratio 1.707) (Chen et al., 2017).

The oral microbiota is the initiating factor of periodontitis and can bridge between periodontitis and other diseases (Kinane et al., 2017; Sanz et al., 2017). For example, Naoyuki found that periodontitis associated with *Porphyromonas gingivalis* can aggravate the pathological characteristics of AD, including promoting the accumulation of Aβ protein and aggravating cognitive impairment. The main mechanism suggested is that local periodontal inflammation can stimulate brain tissue inflammation (Ishida et al., 2017). In addition, studies have found that *P. gingivalis* can be detected in the brain tissue of patients with AD. The study also confirmed that the gingipain proteases of *P. gingivalis* are associated with AD lesions suggesting neurotoxicity. Gingipain inhibitor treatment was shown to effectively prevent bacterial infections in experimental mice, reduce inflammation in the nervous system, and reduce neuronal damage (Dominy et al., 2019).

Another important periodontal pathogen implicated in systemic diseases is *Fusobacterium nucleatum* (*F. nucleatum)* (Tefiku et al., 2020). Previous research has shown that *F. nucleatum* is one of the most common oral microbes associated with pregnancy complications (Han, 2011); *F. nucleatum* can be detected in patients with multiple systemic conditions, including head and neck infections (Brook, 2013; Fischer et al., 2015; Stergiopoulou and Walsh, 2016) and gastrointestinal diseases, such as appendicitis (Swidsinski et al., 2011, 2012; Zhong et al., 2014). Experimental evidence suggests that *F. nucleatum* increases host inflammation and promotes cancer metastasis, immune-evasion and drug resistance (Casasanta et al., 2020). However, only one study has reported a correlation between *F. nucleatum* and AD to date. The results of this study indicate that the concentration of antibodies to *F. nucleatum* in patients’ serum with mild cognitive impairment and/or AD is significantly higher than that in a healthy cohort (Sparks Stein et al., 2012). This cross-sectional clinical study provides evidence for the association, but no mechanism study was proposed.

**FIGURE 1:**
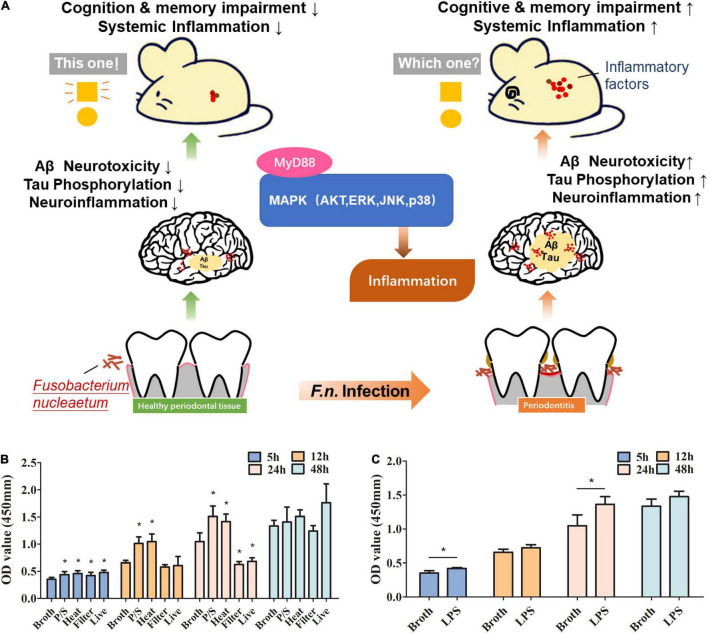
The roles of *F. nucleatum* in promoting proliferation of microglia in a study using an AD animal model. **(A)** Schematic diagram of the pathway that *F. nucleatum* exacerbates Alzheimer’s pathogenesis. **(B)** Evaluation of the effect of *F. nucleatum* has on the proliferation of SIM-A9 cells by CCK8 assay. Bacterial solution treated with the antibiotic, heating, or filtration, along with sterile bacteria broth and untreated bacterial solution were co-cultured with SIM-A9 for 5, 12, 24, and 48 h. **(C)** Evaluation of the effect of LPS of *F. nucleatum* on the proliferation of SIM-A9 cells by CCK8 assay. All data were expressed as mean ± SD.*P < *0.05* vs. Broth group.

In this study, we explore the impact of *F. nucleatum* on AD progression in both *in vitro* experiments and animal models providing the initial evidence of the possible association between a periodontal pathogen and AD and the insight into the underlying mechanisms (Figure 1A).

## Materials and Methods

### Ethical Considerations

This study was conducted in accordance with the policies of Tufts University. All mice used in this study were housed at the Tufts Medical Center Animal Facility (Boston, MA), which is fully accredited by the American Association for Accreditation of Laboratory Animal Care. The Institutional Animal Care and Use Committee (IACUC) approved all animal protocols for this study.

### Co-culture of SIM-A9 Cells and *Fusobacterium nucleatum*

*Fusobacterium nucleatum subsp. nucleatum* (ATCC^®^ 25586*™*) and SIM-A9 microglial cells (ATCC^®^ CRL3265*™*) were obtained from ATCC (Manassas, VA, United States). *Fusobacterium nucleatum* was anaerobically cultured overnight in brain heart infusion broth (Difco, Sparks, MD, United States) containing hemin (0.5%) and vitamin K (0.1%) at 37°C (90% N_2_, 5% H_2_, 5% CO_2_) and adjusted to a concentration of 2 × 10^8^CFU/ml. The experimental groups were designated as follow: **P/S**: *F. nucleatum* was incubated in broth containing 10% penicillin-streptomycin antibiotic mixture (PS, Gibco, Thermo Fisher Scientific, Inc., Waltham, MA, United States) for 2 h at 37°C, the dead cells were centrifugated and resuspended in sterile broth; **Heat:** *F. nucleatum* was incubated in broth for 2 h at 80°C, and the dead cells were centrifugated and resuspended in sterile broth (Liu et al., 2020b); **Filter:** *F. nucleatum* culture supernatant was filtered with a 0.2 μm filter; **Live:** untreated *F. nucleatum* in culture medium. Sterile broth alone was used as a control (**Broth**). SIM-A9 cells were cultured in a 6-well plate or 96-well plate in 5% CO_2_ and 95% air at 37°C in complete growth medium: DMEF/12 medium containing 10% Fetal bovine serum and 5% Horse serum (Gibco, Thermo Fisher Scientific, Inc., Waltham, MA, United States). One percent phosphatidyl serine (PS) was added. When the cells achieved 80% confluence, the medium was changed to complete growth medium without PS and bacteria were added (ratio of bacterial broth and cell medium was 1:10) and co-cultured for different periods for different experiments as indicated (Nagamoto-Combs et al., 2014).

### Cell Proliferation Test

The impact of *F. nucleatum* on SIM-A9 proliferation was assessed using the Cell Counting Kit-8 (CCK-8) (Dojindo Laboratories, Kumamoto, Japan) following the manufacturer’s instructions. After co-culture, cells were treated with 10 % CCK-8 for approximately 2 h. The optical density (OD) value was determined at 450 nm using a microplate reader to calculate cell proliferation.

### *Fusobacterium nucleatum* LPS Extraction

The bacterial suspension (OD600nm = 0.8-1.2) was treated with proteinase K for 30 min at 50°C and LPS was extracted using an LPS extraction kit (iNtRON Biotechnology, United States) following the manufacturer’s instructions.

### RNA Extraction, Reverse Transcription, and Real-Time Quantitative PCR (qRT- PCR)

SIM A-9 cells were collected from plates and total RNA was extracted using a Quick-RNA Miniprep Kit (ZYMO Research, Irvine, CA, United States). 1 μg of total RNA was subjected to reverse transcription using M-MLV Reverse Transcriptase (Thermo Scientific, Waltham, MA, United States) according to the manufacturer’s protocol. For qRT-PCR, PowerUp SYBR Green Master Mix (Thermo Scientific) was used, and experiments conducted on a Bio-Rad iQ5 thermal cycler (Bio-Rad Laboratories, Hercules, CA, United States). Differences in expression were evaluated by the comparative cycle threshold method using GAPDH as a control. To extract total RNA from mouse tissues, TriZol reagent (Life Technologies) was added to homogenized tissue according to the manufacturer’s instructions followed by qRT-PCR assays as we described previously (Qiu et al., 2020).

### Animal Preparation, Experimental Periodontitis, and *Fusobacterium nucleatum* Infection

B6 congenic 5XFAD transgenic (Tg) mice were obtained from Jackson Laboratories (MMRRC Stock No: 34848-JAX, Bar Harbor, ME, United States). 5XFAD transgenic mice overexpress both mutant human amyloid beta (A4) precursor protein 695 (APP) with the Swedish (K670N, M671L), Florida (I716V), and London (V717I) Familial AD (FAD) mutations and human PS1 harboring two FAD mutations, M146L and L286V. The expression of both transgenes is regulated by neural-specific elements of the mouse *Thy1* promoter to drive overexpression in the brain. These mice exhibit plaque deposition at 2 months of age, along with increased microgliosis and astrocytosis (Oakley et al., 2006; Barrett et al., 2019). Wildtype B6 mice were also purchased from Jackson Laboratories (Stock No: 000664, Bar Harbor, ME, United States) as control.

To induce periodontitis, after ligature placement around maxillary second molars (left side) under anesthesia with ketamine and xylazine, 100 μL *F. nucleatum* (OD600nm = 1) suspension was applied topically to the buccal surface of the molars of 4-month-old mice (*n* = 5) every other day for 2 months under anesthesia as we previously described (Wu et al., 2019; Lian et al., 2020). The ligatures were checked every other day, replaced as necessary and kept in place until the end of *F. nucleatum* application. The uninfected group of mice was the same age, and 100 μL of sterile broth was applied to the molars without ligation at the same frequency.

### Mouse Tissue Collection

Two months after induction of periodontitis, the animals were anesthetized with ketamine and xylazine and euthanized by transcranial perfusion with normal saline and 4% paraformaldehyde in phosphate-buffered saline (PBS, 0.1 M, pH 7.4). All the mice survived until the sample collection. The brain was dissected; one hemibrain was fixed in 4% formalin and dehydrated for histological analysis and the other was stored at −80°C for future analyses. Mouse whole blood was collected and immediately centrifuged at 4200 × *g* for 10 min to obtain the plasma for further use.

### Behavioral Evaluations

The novel object recognition task test was performed before, one and two months after induction of periodontitis. The apparatus consisted of a dark open field box (45 cm × 45 cm × 50 cm) according to the method of Lueptow (2017). Ethovision XT software (Noldus, Wageningen, Netherlands) was used to measure the patterns of movement, time spent on each item, velocity and distance traveled. These measurements were used as indicators of exploration.

### Protein Extraction and Western Blot Analysis

Mouse brains were homogenized in liquid nitrogen and protein was extracted using RIPA Lysis and Extraction Buffer containing three protease inhibitors (Thermo Scientific, Waltham, MA, United States). Western blot analyses were performed as previously described (Qiu et al., 2020). Antibodies for glyceraldehyde 3-phosphate dehydrogenase (GAPDH) (cat# 2118,1:1,000), MyD88 (cat# 4283,1:1,000), p-P38 (cat# 9211,1:1,000), P38 (cat# 8690, 1:1,000) and p-Akt (cat# 4060, 1:500), were purchased from Cell Signaling Technology (Danvers, MA, United States), p-JNK (cat# sc-12882, 1:500), JNK (cat# sc-7345, 1:500), p-ERK (cat# sc-7383,1:500), ERK (cat# sc-514,302, 1:500) and Akt (cat# sc-81434, 1:500) were purchased from Santa Cruz Biotechnology, Inc. (Dallas, TX, United States) and antibodies for beta-amyloid (clone 6E10, 1:1,000) were purchased from BioLegend (San Diego, CA, United States). Antibodies for AT8 (cat MN1020, 1:1000) were purchased from Thermo Scientific (Waltham, MA, United States). Blots were visualized using ECL chemiluminescence reagents from Thermo Fisher. All bands were quantitatively analyzed using Image J (ImageJ, RRID: SCR_003070).

### Immunohistochemistry

Hemi-brains were paraffin embedded and 6-μM-thick sections were prepared for IHC. IHC was performed using a Histostain-SP Kit (Life Technologies, Waltham, MA, United States) following the manufacturer’s recommendations. Primary antibody for iba-1 (cat# 17198, 1:2,000) was purchased from Cell Signaling Technology (Danvers, MA, United States), p-tau (cat MN1020, 1:500) was from Thermo Scientific (Waltham, MA, United States)and antibody to beta-amyloid (clone 6E10, 1:5,000) was purchased from BioLegend (San Diego, CA, United States). Digital images of stained tissues were taken with an Olympus BX53 microscope.

### Enzyme-Linked Immunosorbent Assay

Mouse plasma was collected and used for the measurement of TNF-α using ELISA kits (Abcam, Cambridge, United Kingdom) according to the manufacturer’s instructions.

### Quantitative Proteome

Mouse brain samples from infected and uninfected 5XFAD mice (*n* = 3) were sent to Poochon Scientific LLC. (Frederick, Maryland, United States) for Quantitative Proteome analysis using a standardized mass spectrometry-based quantitative proteomic profiling workflow. In brief, the workflow includes the preparation of cell lysate, trypsin digestion, TMT-7plex labeling of tryptic peptides, fractionation of labeled peptides by reverse-phase UHPLC, LC-MS/MS analysis, database search, quantification analysis and data analysis.

### TMT-7plex Labeling

Mouse samples were prepared for lysis and the protein concentration of the lysates was determined using a BCA*™* Reducing Reagent compatible assay kit (Thermo Scientific; Rockford, IL, United States). One TMT-7plex set was used for labeling six samples (Supplementary Table 1) taking 100 μg of protein lysate from each sample for in-solution trypsin digestion. Pre-digestion samples were run along with digested samples on SDS-PAGE and stained to check digestion efficiency. Isobaric labeling was performed using the TMT-7plex kit for six samples (six samples plus one of master mix of equal amount of six digested peptide samples as the reference) according to the product manual (Supplementary Table 1). Each set of TMT labeled peptide mixtures was combined and the labeled peptide mixtures were dried in a vacuum concentrator and kept at −80°C.

### Fractionation of Labeled Peptides by Basic Reverse Phase UHPLC

The dried labeled peptides were resuspended in 10 mM TEABC. The labeling efficiency was determined before fractionation by analysis of a small aliquot of the sample (1%). A minimum of 95% labeling efficiency was required. The fractionation of the TMT-11plex labeled peptide mixture was carried out using an Agilent AdvanceBio Column (2.7 μm, 2.1 × 250 mm) with Solvent A (10 mM TEABC, pH 8.0) and an Agilent UHPLC 1290 system. The separation was performed by running a gradient of Solvent B (10 mM TEABC, pH 8.0, 90% ACN) and Solvent A (10 mM TEABC, pH 8.0) at a flow rate of 250 μL/min. The eluted fractions were collected into a 96-well plate using a 1260 series auto-sample fraction collector. The 96 eluted fractions were further combined into 24 fractions according to collection time (e.g., Ax/Cx/Ex/Gx, and Bx/Dx/Fx/Hx; A/B/C/D/E/F/G/H represents the column, x represents row 1 through row 12) for LC/MS/MS analysis.

### Nanospray LC/MS/MS Analysis and Database Search

The LC/MS/MS analysis was carried out using a Thermo Scientific Q-Exactive hybrid Quadrupole-Orbitrap Mass Spectrometer and a Thermo Dionex UltiMate 3000 RSLCnano System. Each peptide fraction from a set of 24 fractions was loaded into a peptide trap cartridge at a flow rate of 5 μL/min. The trapped peptides were eluted onto a reverse-phase 20 cm C18 PicoFrit column (New Objective, Woburn, MA) using a linear gradient of acetonitrile (3-36%) in 0.1% formic acid. The elution duration was 110 min at a flow rate of 0.3 μL/min. Eluted peptides from the PicoFrit column are ionized and sprayed into the mass spectrometer, using a Nanospray Flex Ion Source ES071 (Thermo, San Jose, CA, United States) at spray voltage 1.8 kV and capillary temperature 250°C. Twenty four fractions were analyzed sequentially.

The Q Exactive instrument was operated in the data-dependent mode to automatically switch between full scan MS and MS/MS acquisition. Survey full scan MS spectra (m/z 350-1800) were acquired in the Orbitrap with 35,000 resolutions (m/z 200) after an accumulation of ions to a 3 × 10^6^ target value based on predictive automatic gain control (AGC). The maximal injection time was set to 100 ms. The 15 most intense multiply charged ions (*z* ≥ 2) were sequentially isolated and fragmented in the octupole collision cell by higher-energy collisional dissociation (HCD) using a normalized HCD collision energy of 30 with an AGC target 1 × 10^5^ and a maximal injection time of 400 ms at 17,500 resolutions. The isolation window was set to 2 and fixed first mass was 120 m/z. The dynamic exclusion was set to 20 s. Charge state screening was enabled to reject unassigned and 1 +, 7 +, 8 +, and > 8 + ions.

One set of 24 MS Raw data files acquired from the analysis of 24 fractions was searched against mouse protein sequence databases obtained from the UniProt KB website using Proteome Discoverer 2.4 software (Thermo, San Jose, CA, United States) based on the SEQUEST and percolator algorithms. The false positive discovery rates (FDR) were set at 1%. The resulting Proteome Discoverer Report contains all assembled proteins with peptide sequences and peptide spectrum match counts (PSM#) and TMT-tag based quantification ratio.

### Data Analysis of the Quantitative Proteome

TMT-tag based quantification was used for determining the relative abundance of proteins identified from six samples using one TMT-7plex. The common reference tag 130C (a mix of six samples) was used for the calculation of the ratio (e.g., sample 1-TMT-tag-126 versus Mix-Tag-130C). The relative abundance of proteins in each TMT-set was normalized using the reference mix. The raw data has been uploaded to the ProteomeXchange Consortium via the PRIDE (Perez-Riverol et al., 2022) partner repository.

### Statistics

All the experiments were designed to generate groups of equal size, using randomization and blinded analysis. The initial sample sizes were estimated using the PASS statistical software package (Number Cruncher Statistical Systems, Kaysville, UT, United States). The significance level (alpha) was set as 0.05 and the power (1-beta) was set as 0.9. The experimental data obtained are expressed as mean ± standard deviation, and SPSS19.0 and GraphPad Prism5.0 software were used for statistical analysis and visualization. The comparison between groups was performed by two-tailed Student’s *t*-test, and the comparison between three or more groups was performed by one-way ANOVA in which Dunnett’s test was used for the pairwise comparison. *P* < *0.05* was considered as a significant difference.

## Results

### *Fusobacterium nucleatum* Promotes Proliferation of Microglia

Microglia account for about 10% of the total number of cells in neuronal tissues. Under normal circumstances, the number of microglia in the nervous system is relatively stable. To explore whether *F. nucleatum* affects the proliferation of microglia, SIM-A9 cells were co-cultured with the bacteria. CCK8 was used to detect changes in the number of microglial cells at 5h, 12h, 24h and 48h after co-cultivation. The results showed that compared with the control group, after 5 h of co-cultivation, all *F. nucleatum* preparations promoted the growth of SIM-A9 cells (*P* < 0.05) (Figure 1B), while after 12 h of co-cultivation, only the P/S treated and heat-treated *F. nucleatum* promoted cell proliferation (P < 0.05); filtered bacterial solution and live bacteria did not significantly promote cell growth. After 24 h, P/S treated, and heat-treated *F. nucleatum* still promoted proliferation of SIM-A9 cells (*P* < 0.05), but the filtered bacterial broth and the live bacteria inhibited the growth of SIM-A9. At 48 h, there was no significant difference between groups (*P* > 0.05).

Lipopolysaccharide is a heat resistant component of cell wall and an important virulence factor of gram-negative bacteria (Dominy et al., 2019). P/S treated, and heat-treated bacteria promoted the growth of SIM-A9 cells implicating LPS as a key factor. To determine the impact of *F. nucleatum* LPS on microglial proliferation, LPS was extracted and added to the SIM-A9 cell culture medium. The results reveal that LPS promoted the proliferation of brain microglia (Figure 1C), which was significant at 5h and 24h (P < 0.05). At 12h and 48h, there was non-significant promotion of proliferation.

### *Fusobacterium nucleatum* Activate Microglia Activity *in vitro*

**FIGURE 2:**
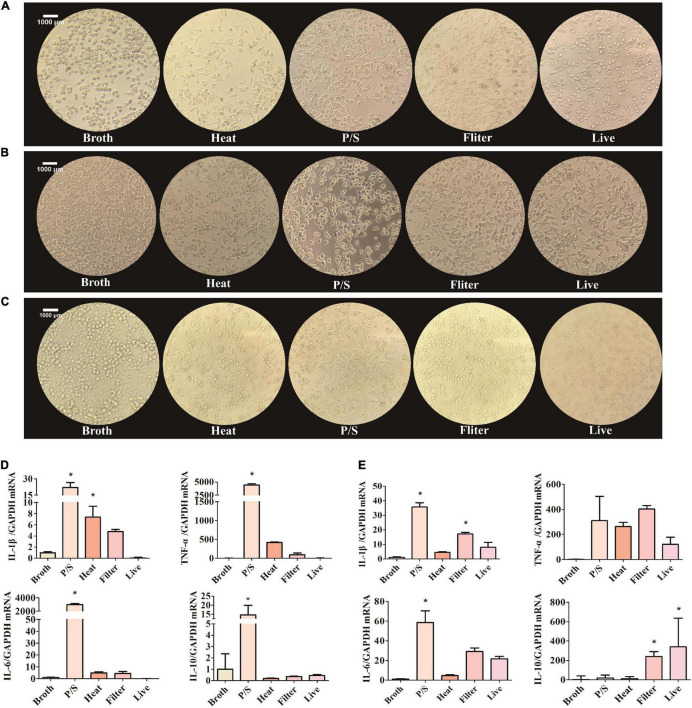
*F. nucleatum* could activate the SIM-A9 cells *in vitro.* **(A–C)** The morphological changes of SIM-A9 cells after co-culturing with *F. nucleatum* for **(A)** 5 h, **(B)** 24 h and **(C)** 48 h. **(D,E)** The expression level of genes of inflammatory factors from SIM-A9 after co-culturing with *F. nucleatum* for **(D)** 5 h and **(E)** 12 h. All data were expressed as mean ± SD. *P < *0.05* vs. Broth group.

When stimulated, microglia change morphology from ramified to polygon or amoeboid. To explore whether *F. nucleatum* influenced the morphology of SIM-A9 cells, pictures of the cell morphology were captured after co-cultivation for 5, 24, and 48 h. After cells were co-cultured with bacteria, cell morphology changed significantly. Cell volume increased and assumed a rod-shaped or amoeba-like appearance (Figures 2A–C). However, in the control group, no obvious morphological changes occurred.

Another typical phenotypic change of microglia in response to external stimuli is the secretion of inflammatory molecules. To detect the expression of inflammatory factors by SIM-A9 cells after co-cultivation with *F. nucleatum*, RNA was extracted from cells and RT-qPCR was used to detect the expression of mRNA of inflammatory genes. After 5 h of co-culture, the *F. nucleatum* treated with P/S group significantly promoted the expression of IL-1β, TNF-α, IL-6 and IL −10 mRNA by SIM-A9 cells (*P* < 0.05) (Figure 2D). Although heat-treated and filtered bacterial culture medium of *F. nucleatum* also increased the expression of inflammatory factor genes, the results were not statistically significant. Live bacteria had no obvious effect on the inflammatory response.

Similar results were obtained after 12 h of co-culture. The gene expression levels of IL-1β, TNF-α and IL-6 by SIM-A9 cells in the P/S-treated *F. nucleatum* group increased significantly (P < 0.05) (Figure 2E), but the level of IL-10 did not change significantly (*P* > 0.05). When microglia were cultured with filtered bacterial culture medium for 12 h, the gene expression of IL-1β, TNF-α, IL-6 and IL-10 were elevated; only IL-1β and IL-10 were statistically significant (*P* < 0.05).

### *Fusobacterium nucleatum* Exacerbates Behavioral Phenotypical Changes in 5XFAD Mice

**FIGURE 3:**
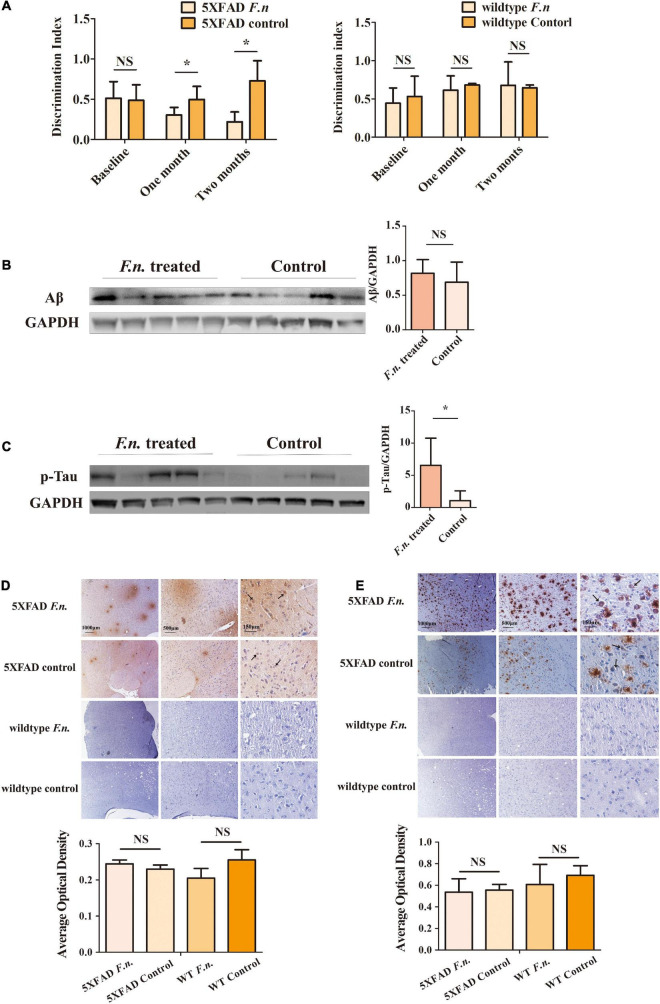
The behavioral and pathological manifestation of 6-month-old 5XFAD mice and 6-month-old wild-type mice with or without *F. nucleatum* infection. **(A)** Results of novel object recognition (NOR) test of mice at baseline, one month and two months after infection. **(B,C)** The expression levels of **(B)** Aβ and **(C)** Tau protein in mice brain after two months of infection with *F. nucleatum*. **(D,E)** The distribution of **(D)** Aβ and **(E)** Tau protein in mice cerebrum cortex after two months of infection. All data were expressed as mean ± SD, *n* = 5, *P < *0.05* vs. the control group. NA, not significantly different.

To determine the clinical consequences of *F. nucleatum* infection, the novel object recognition task test was employed. Results revealed that the infected group of mice exhibited a lower discrimination index compared to controls, especially one month after the treatment. Mice infected with *F. nucleatum* failed to differentiate between a novel object and a familiar object, which suggests that the *F. nucleatum* treated mice suffered from a more serious memory impairment than the control 5XFAD mice (Figure 3A). No significant differences were seen between any conditions in control wild type B6 mice (Figure 3A). The results indicate that in 5XFAD mice, memory impairment was enhanced by *F. nucleatum* infection.

### *Fusobacterium nucleatum* Enhances the Deposition of Tau Protein and Beta-Amyloid

In addition to behavioral testing, biological manifestations of *F. nucleatum* treatment in the brain were also determined. After two-months of infection with *F. nucleatum*, the brain tissues of the mice were collected and protein was extracted. The amount of beta-amyloid and of p-Tau protein in the brain tissue were detected (Jeon et al., 2020; Delizannis et al., 2021). The results of western blot analysis showed there was a slightly higher expression of beta-amyloid in infected 5XFAD mice compared to non-treated 5XFAD mice, but the results were not statistically significant (*P* > 0.05) (Figure 3B). The amount of p-Tau protein in the brain tissue of the treated group was significantly higher than that of the control group (*P* < 0.05) (Figure 3C).

Immunohistochemical staining also showed a wider distribution of Tau protein and beta-amyloid plaques in *F. nucleatum* infected 5XFAD mice (Figures 3D,E, black arrows) than the non-treated group. Neither Tau protein nor beta-amyloid was detectable in control wild type B6 mice. The above results suggest that *F. nucleatum* might play a significant role in exacerbating the behavioral and biological manifestations of 5XFAD mice, promoting the development of the disease.

### *Fusobacterium nucleatum* Infection Increases Inflammation in a Mouse AD Model

**FIGURE 4:**
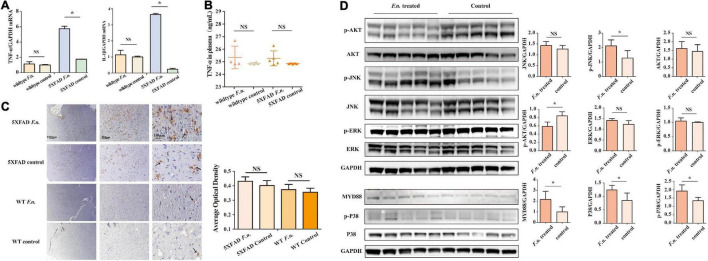
*F. nucleatum* could promote inflammatory processes in 6-month-old 5XFAD mice brain. **(A)** The expression levels of genes of *TNF*-α and *IL*-β in the brain tissue of 5XFAD mice and wild-type mice. **(B)** The concentration of TNF-α in mice plasma. **(C)** The distribution of microglia in mice cerebrum cortex. **(D)** The expression levels of proteins in inflammation-related pathways in 5XFAD mice brain. All data were expressed in mean ± SD, *n* = 5, *P < *0.05* vs. the control group. NA, not significantly different.

Inflammation is a driving factor for the development of AD. To determine whether *F. nucleatum* influences systemic and local inflammation in 5XFAD and wild-type mice, the expression of TNF-α and IL-β genes in brain tissue was assessed by RT qPCR (Figure 4A), and in plasma, TNF-α levels were determined by ELISA. The results revealed that in 5XFAD mice, the expression of TNF-α and IL-β genes in the brain was significantly increased in mice with *F. nucleatum* infection (P < 0.05). The expression of TNF-α and IL-β genes was approximately 3 times higher than in the uninfected group. In wild-type mice, no significant differences were seen between groups after 2 months of infection (P > 0.05). The concentration of plasma TNF-α in the *F. nucleatum* treated group was not significantly different from the uninfected group (Figure 4B).

### Activation of Inflammation Pathways by *Fusobacterium nucleatum* Infection

The distribution of microglia in the mouse brain was determined by immunohistochemical staining (Figure 4C), after brain sections were stained with iba1. The results show a wider distribution of microglia in the brain of 5XFAD mice with infection than their non-infected counterparts, indicating that the proliferation and migration of microglia in the brain was increased. However, in control wild type mice B6, few microglia were detected in the brain and *F. nucleatum* did not have any impact on the distribution of microglia.

The relevant signaling pathways of inflammation in 5XFAD mouse brains were determined by western blot (Figure 4D). The results revealed that MyD88 protein in the brains of infected mice was higher (P < 0.05) than non-infected group. Likewise, the expression of P38 protein was significantly increased as was phosphorylated P38 protein (*P* < 0.05). Moreover, the results also show that *F. nucleatum* stimulated the JNK pathway; the expression levels of p-JNK and JNK proteins were raised. Among them, phosphorylated JNK increased significantly (*P* < 0.05), while the level of JNK increased slightly (*P* > 0.05). The expression of Akt and ERK proteins was not influenced by *F. nucleatum* (*P* > 0.05).

### *Fusobacterium nucleatum* Infection Induces Specific Protein Expression in Mouse Brain

**FIGURE 5:**
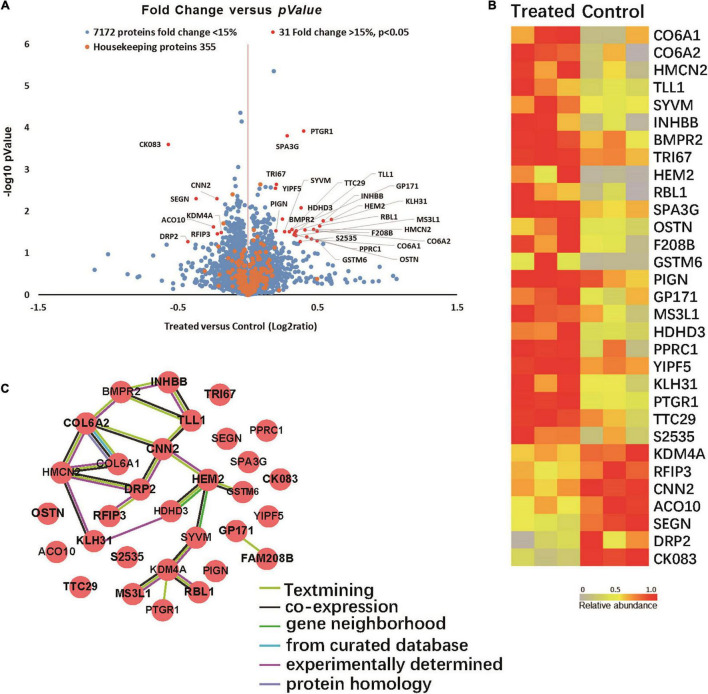
**(A)** Volcano plot demonstrating the fold change of 7558 protein abundance between treated group and control group (*n* = 3). Volcano plot demonstrating the fold change of 7558 protein abundance between treated group and control group (*n* = 3). The *x*-axis represents the log2 of fold changes (treated versus control), and the y-axis represents the statistically significant *p*-value (–log10 of *p*-value, *n* = 3). Blue dots represent 7172 protein fold change <1.15, Red dots are 31 proteins fold change >1.15, *p* > 0.05, and Orange dots represent 355 housekeeping proteins. **(B)** Proteomic characterization of the proteomes of 6 mice brain tissue samples by TMT-7plex labeling based quantitative proteomics. A heat map showing the relative abundance of 31 ranked proteins (Ratio of Treated to Control ≥ 1.15 or ≤ 0.87, *p* < 0.05, *n* = 3) was identified across two groups of 6 samples. The color key indicates the relative abundance of each protein (0 to 1.0) across 6 samples. **(C)** Protein-protein interaction networks. (31 items (mouse) - STRING interaction network (string-db.org)).

Quantitative proteomic analyses were performed to detect proteins in the brains of two groups of 5XFAD mice. Total of 7,558 proteins were found in all samples (Figure 5A). Among them 31 proteins were significantly up- or down-regulated by at least 15% in treated groups compared to control group (P < 0.05). Specifically, 24 proteins were up-regulated (Ratio (treated/control) > 1.15, P < 0.05) in the treated groups and 7 were down-regulated (Ratio (treated/control) < 0.87, P < 0.05).

Among the proteins with significant changes in expression (Figure 5B), GSTM6, RBL1, SPA3G and PTGR1 were upregulated in the treated group. These changes correlated with glutathione transferase activity, p53 pathway feedback loop 2, the apoptotic process and response to toxic substance pathways. SEGN and DRP2 were downregulated in the infected group. SEGN and DRP2 are involved in central nervous system development and calcium ion binding pathways.

The protein-protein interactions of the 31 differentially expressed proteins were analyzed *via* the String website. Figure 5C shows that COLA1, COLA2, HMCN2, and HEM2 proteins interact with more protein nodes, which might suggest their central role in this interaction network that can play an important role in the progression of AD.

## Discussion

Microglia are the major immune cells in the nervous system (Bagheri-Mohammadi, 2020) that are activated by abnormal signals or exogenous stimuli, such as LPS and Aβ. It is well known that microglia could be categorized into two types: the M1 phenotype and M2 phenotype. The stimnuli like LPS, Aβ or IFN-γ would activate M1 phenotype, leading to the expression of pro-inflammatory cytokines and irreversible neuron loss. The M2 phenotype could be due to the exposure of IL-4 or IL-13, resulting in resolution of inflammation and tissue repair (Orihuela et al., 2016; Tang and Le, 2016).

Microglia reside in the brain parenchyma and do not circulate, but they proliferate in a large quantity when being activated to regulate the inflammatory response (Crotti and Ransohoff, 2016). In healthy brain, microglia could help maintain the stability of the nervous system. They can impact the function of astrocytes and neurons by expressing soluble molecules, help remove cell debris and polymerized proteins, and they also play an active role in synaptic pruning (Paolicelli et al., 2011). There is evidence that the activation of microglia is related to many neurological diseases or conditions, such as brain injury, ischemic stroke, and neuroinflammatory diseases. Due to the close relationship between activation of microglia and inflammation in the nervous system (Araki et al., 2020; Bourgognon and Cavanagh, 2020), it is generally accepted that the activation of microglia is closely related to the pathogenesis of neurodegenerative diseases, such as AD and Parkinson’s disease (Ashford et al., 2020; Stolero and Frenkel, 2020; Forloni et al., 2021).

This report demonstrates that *F. nucleatum* promotes the proliferation of microglia. The increase in cell mass is an important indicator of microglial activation (Crotti and Ransohoff, 2016). Promoting proliferation was observed in short-term co-culture; after 5, 12, and 24 h, but not in 48 h suggesting a rapid cellular response to challenge. As microglial cells proliferate, inflammatory immune function increases resulting in changes in cell morphology and the increased expression of inflammatory genes.

The main known virulence factors of *F. nucleatum* include FadA, Fap2, and LPS (Han, 2015), which possess unique characteristics. We employed a series of pretreatments to *F. nucleatum* to systematically inactivate these various virulence factors and determined that LPS is a one of the candidates for microglial activation. For example, antibiotics kill bacteria or limit growth, but do not affect bacterial virulence factors on the cell surface; heating denatures bacterial surface proteins, but not LPS. Filtration removes bacterial cells leaving metabolites in the culture medium, which might contain exosomes, secreted products or bacterial fragments. Interestingly, live bacteria reduced SIM-A9 cell viability after 5 h *in vitro*, thus while we were able to assess the impact of various virulence factors in longer experiments, the differences in proliferation and inflammatory responses with live bacteria could not be assessed after 5 h.

The results obtained with these different treatments suggest that LPS is one of the possible candidates for microglial activation. There are previous reports supporting that LPS is an important factor leading to inflammation in the nervous system (Catorce and Gevorkian, 2016). We further explored this pathway using pure LPS extracted from *F. nucleatum* and demonstrate that LPS from *F. nucleatum* promotes the proliferation of SIM-A9, which was in line with expectations. However, LPS is just one of the many virulence factors of *F. nucleatum*. Whether other heat resistant virulence factors activate microglia remains to be explored. For example, previous study reported that the virulence factor FadA secreted by *F. nucleatum* promotes both periodontal bone loss and colorectal tumorigenesis (Meng et al., 2021). This occurs when FadA transforms into an amyloid-like structure converting *F. nucleatum* from a benign commensal to a virulent pathogen. Since amyloid proteins are also heat resistant, it could be possible that amyloid FadA may be involved in microglial activation. The effects of amyloid FadA on microglia cells and Alzheimer’s Disease warrant further examination.

Apart from *in vitro* experiments, the impact of *F. nucleatum* on mouse pathological manifestations was also determined *in vivo*. A mouse oral infection model was established by applying bacterial solution topically to the buccal surface of the maxillary mucosa and gingiva of ligatured teeth. A review of previous literature revealed that it could take 22 weeks of chronic oral topical bacteria application to induce brain inflammation (Ilievski et al., 2018). Thus, to ensure the attachment and colonization of *F. nucleatum* and establish a rapid infection model, the mouse maxillary left second molar was ligated with silk thread. Two months later, periodontitis was confirmed with the changes that height of the alveolar bone in infected mice was decreased by micro-CT scanning (Supplementary Figure 1). Previous studies also showed that even without ligature, *F. nucleatum* induced periodontal bone loss in an FadA-depedent manner after ten weeks of oral inoculation (Meng et al., 2021).

As shown in the results, *F. nucleatum* exacerbates pathological and behavioral manifestations in 5XFAD mice. Compared with uninfected 5XFAD mice, infected mice performed poorly in the novel object recognition task test (spent less time exploring novel items during the testing phase). In addition, the amount of Aβ and p-Tau protein in mouse brain tissue was significantly higher than that of the untreated group suggesting that *F. nucleatum* exacerbates pathological and behavioral manifestations in 5XFAD mice. We have conducted the PCR to identify the presence of *F. nucleatum* in brain tissue but the result was negative, which imply that *F. nucleatum* may induce inflammatory responses without actually colonizing the brain or the amount of bacteria in brain was beblow the limit of the detection. Furthermore, it is well-known that the gram-negative endotoxin, namely lipopolysaccharide (LPS), could induce blood-brain barrier (BBB) disruption (Banks et al., 2015), leading to altered permeability or breakdown of the BBB. Thus, the BBB injury could happen and affect the onset and progression of AD (Sweeney et al., 2019; Yang et al., 2021). In the present study, the BBB breakdown might happen due to the LPS of *F. nucleatum* and consequently result in exacerbation of pathological and behavioral manifestations of 5XFAD mice. This might explain the corresponding changes in symptoms even without the detection of *F. nucleatum* in the brain tissue.

Previous studies suggested that local inflammation in the central nervous system leads to cognitive impairment. For example, the inflammation in the brain of AD patients is increased (Teixeira et al., 2008). Inflammatory cytokines are released, including interleukin family proteins, TNF-α, TGF-β and chemokines, which may be used as serum and plasma markers in AD (Lee et al., 2009). The over-activation of microglia is an early feature of AD, and TNF-α is a pro-inflammatory factor mainly produced by activated microglia/macrophages, which could play a central role in the mechanism of the onset of AD (Perry et al., 2001). Many clinical and animal studies have shown that there is a link between excessive TNF-α in the brain and AD (Akiyama et al., 2000; Tarkowski et al., 2003). Excessive TNF-α in brain tissue disrupts Aβ clearance mediated by brain microglia (Koenigsknecht-Talboo and Landreth, 2005) resulting in increased Aβ protein accumulation (Liao et al., 2004; Yamamoto et al., 2007), causing synaptic dysfunction (Ralay Ranaivo et al., 2006), thereby speeding up disease development and cognitive impairment (McGeer and McGeer, 2003).

Another pro-inflammatory factor that is essential in AD is IL-1β. It is known that IL-1β is a key regulator of acute inflammation in the central nervous system. Neuroinflammation caused by IL-1β is also related to the pathophysiological processes of chronic neurodegenerative diseases, including AD. Previous studies have suggested that the deposition of Aβ in the brain causes microglia to secrete IL-1β, which leads to chronic neuroinflammation in AD, which causes neuronal dysfunction and ultimately accelerates the process of neurodegeneration (Teixeira et al., 2008; Lai et al., 2017). IL-1β also enhances the synthesis of Aβ protein precursor mRNA in human endothelial cells (Goldgaber et al., 1989) suggesting that IL-1β also influences the formation of senile plaques.

Several studies investigating AD-related inflammation have determined the levels of TNF-α and IL-1β in brain tissue (Yasutake et al., 2006; Li et al., 2017; Saffari et al., 2020) confirming the close connection between the two and AD. Here, we focused on the change in TNF-α and IL-1β message levels in the brain tissue of mice after infection with *F. nucleatum*. The results of qRT-PCR showed that, in 5XFAD mice, the expression levels of TNF-α and IL-1β mRNA are elevated after infection with *F. nucleatum*. Elevations of TNF-α and IL-1β were not detected in serum and plasma. Subsequently, the expression of proteins related to inflammation signaling pathways in mouse brain tissue was assessed. The adapter protein MyD88 plays a pivotal role in LPS stimulated pro-inflammatory signaling pathways and P38 is known to contribute to the LPS-stimulated inflammatory response (Qiu et al., 2020). Here, we showed that in the presence of *F. nucleatum* infection, the expression of P38 protein in 5XFAD mouse brain tissue was significantly upregulated, as was its phosphorylation level. MyD88 protein was upregulated. The results of immunohistochemical staining revealed a significant increase in the numbers of microglia in the brains of 5XFAD mice treated with *F. nucleatum*. The actions of these potent cytokines appear to be local as no increases in TNF-α or IL-1β were noted in plasma. Together the data suggest that local overgrowth of bacteria induced by periodontitis could be transported to the brain and lead to local inflammation, thereby aggravating AD.

Quantitative proteomics refers to the mass spectrometric detection of specific known proteins (Doerr, 2013). Proteomic studies were performed to compare the similarities and differences in protein expression by microglial cells under different physiological or pathological conditions, and to classify and identify related proteins. More importantly, proteomics can be used to analyze the interactions between proteins, and the function of individual proteins. We used quantitative proteomics technology to detect the proteins in the brain tissues of 5XFAD with or without *F. nucleatum* infection and found 31 proteins that were significantly differentially expressed by the two groups of mice. The results of quantitative proteomics will be further verified and explored to find key proteins that act in key roles in signaling pathways and to further elucidate the mechanism of *F. nucleatum* actions in AD in future studies.

## Conclusion

In summary, *F. nucleatum* activates microglia cells *in vitro* promoting proliferation and increased inflammatory response. *In vivo*, in a mouse model of periodontitis, *F. nucleatum* accelerates the development of disease by promoting inflammatory responses in the brain, exacerbating the behavioral and pathological manifestations of the 5XFAD mice. As a periodontal pathogen, *F. nucleatum* accelerates the development of AD. These findings provide a theoretical basis for the study of the relationship between *F. nucleatum* and AD and lay a preliminary foundation for further exploring the mechanism of *F. nucleatum* impact on the onset and development of AD.

## Data Availability Statement

The datasets presented in this study can be found in online repositories. The names of the repository/repositories and accession number(s) can be found below: The mass spectrometry proteomics data have been deposited to the ProteomeXchange Consortium *via* the PRIDE (Perez-Riverol et al., 2022) partner repository with the dataset identifier PXD033147.

## Ethics Statement

The animal study was reviewed and approved by Tufts Institutional Animal Care and Use Committee (IACUC).

## Author Contributions

HW and WQ contributed to the conception, design, data acquisition, analysis, and interpretation, drafted and critically revised the manuscript. XZ and XL contributed to the design, data acquisition and analysis, and drafted and revised the manuscript. ZX, IC, AD, TV, YH, NK, and QT contributed to the design and critically revised the manuscript. LC and JC contributed to the conception, design, data analysis, and interpretation, drafted and critically revised the manuscript. All authors gave final approval and agreed to be accountable for all aspects of the work.

## Conflict of Interest

The authors declare that the research was conducted in the absence of any commercial or financial relationships that could be construed as a potential conflict of interest.

## Publisher’s Note

All claims expressed in this article are solely those of the authors and do not necessarily represent those of their affiliated organizations, or those of the publisher, the editors and the reviewers. Any product that may be evaluated in this article, or claim that may be made by its manufacturer, is not guaranteed or endorsed by the publisher.
