# Supplementary material for: The Periodontal Pathogen Fusobacterium nucleatum Exacerbates Alzheimer’s Pathogenesis via Specific Pathways
Source: Front Aging Neurosci. 2022 Jun 23;14:912709. doi: 10.3389/fnagi.2022.912709 (PMC9260256; doi:10.3389/fnagi.2022.912709)
Supplement: Supplementary file 1 [file Table_1.DOCX]

Supplementary Material

The Periodontal Pathogen *Fusobacterium nucleatum* Exacerbates Alzheimer’s Pathogenesis via Specific Pathways

**Hongle Wu^1,2,3, §^, Wei Qiu^4, §^, Xiaofang Zhu^3,5^, Xiangfen Li^2^, Zhongcong Xie^6^, Isabel Carreras^7^, Alpaslan Dedeoglu^8^, Thomas Van Dyke^9^, Yiping W. Han^10^, Nadeem Karimbux^5^,**

**Qisheng Tu^3,5^, Lei Cheng^2, *^ and Jake Chen^3,5,11, *^**

^1^ Department of Endodontics, Stomatological Hospital, Southern Medical University, Guangzhou, China

^2^ State Key Laboratory of Oral Disease, West China Hospital of Stomatology, National Clinical Research Center for Oral Diseases, Sichuan University, Chengdu, China

^3^ Division of Oral Biology, Tufts University School of Dental Medicine, Boston, Massachusetts, USA

^4^ Department of Stomatology, Nanfang Hospital, Southern Medical University, Guangzhou, China

^5^ Department of Periodontology, Tufts University School of Dental Medicine, Boston, Massachusetts, USA

^6^ Geriatric Anesthesia Research Unit, Department of Anesthesia, Critical Care and Pain Medicine, Massachusetts General Hospital and Harvard Medical School, Charlestown, Massachusetts, USA

^7^ Department of Veterans Affairs, VA Boston Healthcare System, Boston, MA; Department of Neurology and Department of Biochemistry School of Medicine, Boston University, Boston, Massachusetts, USA

^8^ Department of Veterans Affairs, VA Boston Healthcare System, Boston, MA; Department of Neurology School of Medicine, Boston University, Boston, Massachusetts, USA

^9^ Clinical and Translational Research, Forsyth Institute, Cambridge, MA; Oral Medicine, Infection, and Immunity, Harvard School of Dental Medicine, Boston, Massachusetts, USA

^10^ Section of Oral, Diagnostic and Rehabilitation Sciences, College of Dental Medicine; Department of Microbiology & Immunology, Vagelos College of Physicians & Surgeons, Columbia University Irvign Medical Center, New York City, New York, USA

^11^ Dept. of Developmental, Molecular and Chemical Biology, Tufts School of Medicine; 
Graduate School of Biomedical Sciences, Tufts University, Boston, Massachusetts, USA

§ Contributed equally

*** Correspondence:**

**Lei Cheng：**chenglei@scu.edu.cn

**Jake Chen:** jk.chen@tufts.edu


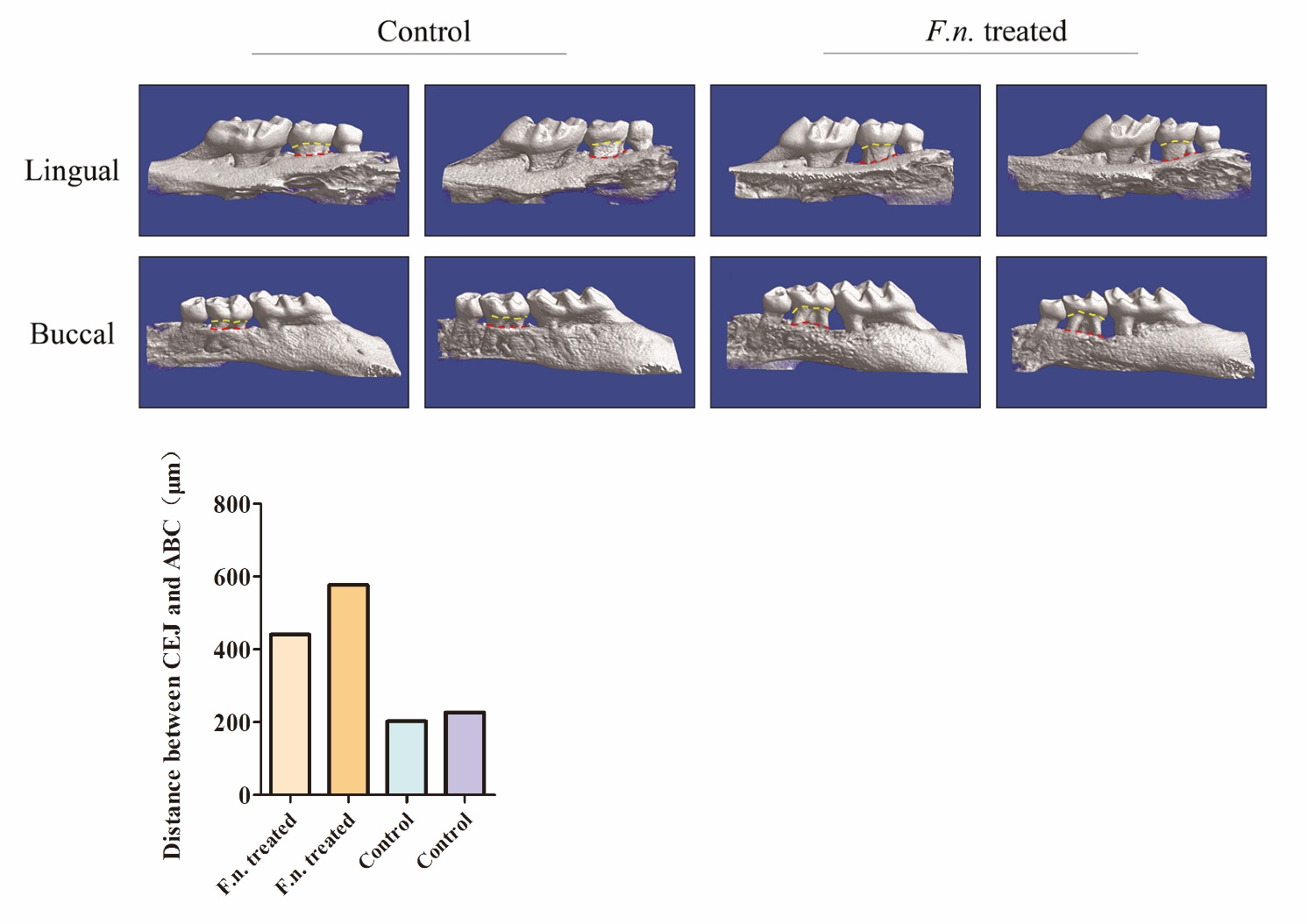


**Supplementary Figure 1.** The results of MicroCT showed the difference in the height of alveolar bone reduction between the *Fusobacterium nucleatum* infection group and the control group. The histogram shows the average distance between the enamel-dentin junction（CEJ） and the alveolar bone crest（ABC） for each mouse.

**Supplementary Table 1: Summary of protein content determined by BCA kit and TMT-labeling naming**

| ID | Conc(mg/ml) | Group | TMT 7plex Tag |
| --- | --- | --- | --- |
| VC1166 | 12.14 | Infected mouse | 126 |
| VC1167 | 12.46 | Infected mouse | 127N |
| VC1168 | 15.86 | Infected mouse | 127C |
| VC1169 | 12.48 | Control | 128N |
| VC1170 | 15.28 | Control | 128C |
| VC1171 | 14.67 | Control | 129N |
| Mix of above 6 samples with equal amount |  |  | 130C |
